# Social Distancing Associations with COVID-19 Infection and Mortality Are Modified by Crowding and Socioeconomic Status

**DOI:** 10.3390/ijerph18094680

**Published:** 2021-04-28

**Authors:** Trang VoPham, Matthew D. Weaver, Gary Adamkiewicz, Jaime E. Hart

**Affiliations:** 1 Epidemiology Program, Fred Hutchinson Cancer Research Center, Division of Public Health Sciences, Seattle, WA 98109, USA; tvopham@fredhutch.org; 2 Department of Epidemiology, University of Washington School of Public Health, Seattle, WA 98195, USA; 3 Departments of Medicine and Neurology, Division of Sleep and Circadian Disorders, Brigham and Women’s Hospital, Boston, MA 02115, USA; mdweaver@bwh.harvard.edu; 4 Division of Sleep Medicine, Harvard Medical School, Boston, MA 02115, USA; 5 Department of Environmental Health, Harvard T.H. Chan School of Public Health, Boston, MA 02115, USA; gadamkie@hsph.harvard.edu; 6 Department of Medicine, Channing Division of Network Medicine, Brigham and Women’s Hospital and Harvard Medical School, Boston, MA 02115, USA

**Keywords:** COVID-19, social distancing, socioeconomic status, household crowding, ecologic study

## Abstract

The SARS-CoV-2 virus is a public health emergency. Social distancing is a key approach to slowing disease transmission. However, more evidence is needed on its efficacy, and little is known on the types of areas where it is more or less effective. We obtained county-level data on COVID-19 incidence and mortality during the first wave, smartphone-based average social distancing (0–5, where higher numbers indicate more social distancing), and census data on demographics and socioeconomic status. Using generalized linear mixed models with a Poisson distribution, we modeled associations between social distancing and COVID-19 incidence and mortality, and multiplicative interaction terms to assess effect modification. In multivariable models, each unit increase in social distancing was associated with a 26% decrease (*p* < 0.0001) in COVID-19 incidence and a 31% decrease (*p* < 0.0001) in COVID-19 mortality. Percent crowding, minority population, and median household income were all statistically significant effect modifiers. County-level increases in social distancing led to reductions in COVID-19 incidence and mortality but were most effective in counties with lower percentages of black residents, higher median household incomes, and with lower levels of household crowding.

## 1. Introduction

The Severe Acute Respiratory Syndrome Coronavirus 2 (SARS-CoV-2) virus has emerged as a worldwide public health emergency. By the beginning of February 2021, more than 105 million cases of COVID-19 had been documented worldwide, and over 2.3 million deaths had been recorded [1]. Although vaccinations have begun, social distancing remains one of the primary public health approaches to slowing disease transmission in the United States and other parts of the world [1]. Theoretically, increases in social distancing—through prohibiting social gatherings, closing non-essential business, and ordering people to stay at home—may reduce disease incidence [2,3], but it cannot be implemented without personal and economic consequences. In a study of 134 countries, lockdowns devised to increase social distancing were shown to reduce COVID-19 transmission. The same study observed proportional decreases in Google mobility metrics and COVID-19 transmission rates [4]. A study modeling the impacts of social distancing in the 25 counties in the United States with the highest numbers of confirmed cases as of 16 April 2020 demonstrated that increases in cellphone-measured social distancing, even before stay-at-home orders, were correlated with a decreased growth in positive COVID-19 test rates [5]. Compelling evidence suggests that in the United States there are clear disparities in disease burden, with higher rates of death from COVID-19 among racial and ethnic minorities [6,7,8]. For example, housing attributes are widely known to be associated with infectious disease through various processes, such as through the transmission of disease between occupants in crowded conditions [9]. The pandemic has already amplified and underscored various gaps in systemic preparedness, as well as a wide range of societal disparities related to race, ethnicity, and socioeconomic status. These disparities are likely driven by numerous risk factors that are independently and jointly concentrated among low-income individuals and families and within communities of color. Health information, beliefs, and behaviors regarding COVID-19 vary by race, ethnicity, and age [10]. However, some factors are removed from individual agency. For example, the ability to comply with social distancing is directly related to numerous correlates of socioeconomic status including occupation, neighborhood attributes, mode of transportation, housing, and household conditions. It is not only the condition of these spaces but the flexibility to re-craft and adapt daily life to a new situation without disruption that is a hallmark of privilege. In numerous reports, COVID-19 mortality rates have been elevated among United States. residents living in the most disadvantaged counties [4,5,6,7].

The effectiveness of social distancing interventions on morbidity and mortality in the United States has not been fully described [5], and their efficacy in different communities is unknown [11]. This evidence is critical to inform public policy decisions such as plans for reopening non-essential businesses, and for future pandemic management [12]. We sought to use data from the first wave of COVID-19 to examine the associations between objective social distancing and COVID-19 incidence and mortality, and to determine if these associations differed in counties with different sociodemographic characteristics and levels of household crowding.

## 2. Materials and Methods

**Figure 1:**
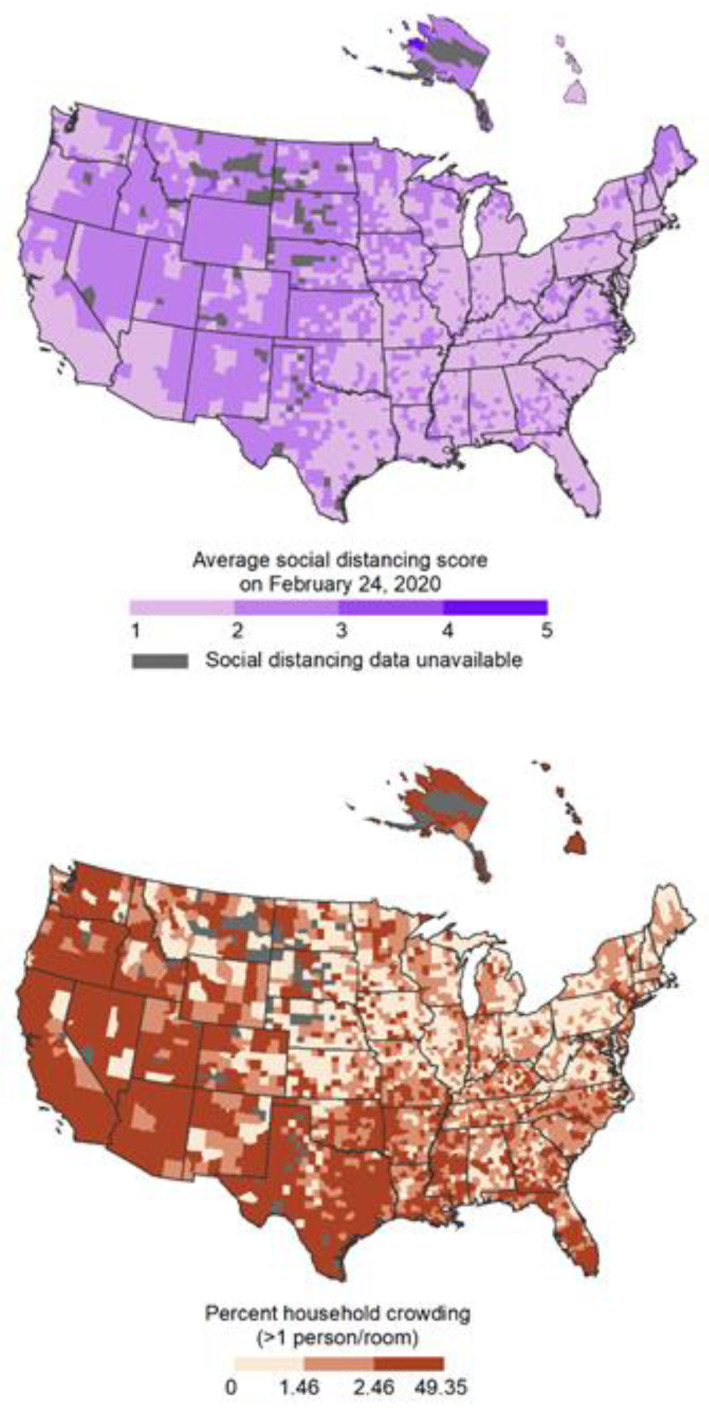
County-level objective social distancing and percent household crowding in the U.S. Social distancing was mapped based on the Unacast scoring scheme and household crowding was mapped using tertiles based on all 3054 counties in the analysis.

Incidence and mortality counts per county through 29 April 2020 (to represent the time period before social distancing policies throughout the United States began to be removed) were collected from the COVID Tracking Project [13]. To determine objective social distancing for each county in the United States, we used nationwide, de-identified smartphone GPS data provided by Unacast (Figure 1) [14]. For this analysis, we utilized data from 24 February 2020 to represent pre-COVID-19 levels of distancing. Objective social distancing, scored 0–5 (5 indicates increased distancing), was calculated based on (1) change in average distance traveled, (2) change in non-essential venue visitation, and (3) the probability that two users were in close proximity [14,15,16,17]. Unacast measures were shown to be correlated with Census data according to geography, income, age, and sex. Covariate data at the county or state level were obtained from the 2018 American Community Survey 5-year estimates, 2019 Robert Wood Johnson Foundation and University of Wisconsin Population Health Institute County Health Rankings, and The Atlantic COVID Tracking Project.

Generalized linear mixed models with a Poisson distribution accounting for counties nested within states were used to calculate incidence rate ratios (IRRs) and 95% confidence intervals (CIs) for a 1-unit increase in objective social distancing. Restricted cubic regression splines were used to test for deviations from linearity. Multivariable models were a priori adjusted for variables associated with incidence rates [18], case ascertainment, or physical features likely to impact distancing, including: county-level Hispanic ethnicity, minority race (includes non-Hispanic black, non-Hispanic Asian, non-Hispanic American Indian or Alaska Native, non-Hispanic Native Hawaiian or Other Pacific Islander, non-Hispanic other or two or more races), percent aged 50 years and older, percent males, median household income, population density, obesity prevalence, percent household crowding (>1 individuals/room) or percent extreme crowding (>1.5 individuals/room), and state-level cumulative COVID-19 testing rate. To determine if the associations between objective social distancing and COVID-19 incidence or mortality were modified, we used multiplicative interaction terms to determine statistical significance and present results stratified by each potential effect modifier. We examined modification by tertiles of county racial and ethnic composition, percent of the county over age 50 years, median household income, percent crowding, and percent extreme crowding. In a sensitivity analyses, we examined the alternate time windows of objective social distancing based on the published incubation periods and symptom onset windows (e.g., 5-day lag, 14-day lag [19]) to determine if the results were robust to our choice to use baseline exposures and scaled Poisson models based on the Pearson and deviance methods accounting for overdispersion. All tests were two-sided and *p* < 0.05 was considered statistically significant. Our study did not constitute human subjects research and was considered exempt from Institutional Review Board review.

## 3. Results

Objective social distancing data were available for 3054 counties (94%) in all 50 states and Washington, D.C. In multivariable-adjusted models, each unit increase in objective social distancing was associated with a 26% decrease in COVID-19 incidence and a 31% decrease in COVID-19 mortality (Table 1). Models were similar regardless of adjustment for percent crowding or percent extreme crowding. Percent crowding, minority population, percent of individuals over 50 years of age, and median household income were all statistically significant modifiers of the associations between objective social distancing and COVID-19 incidence and mortality (Table 2). In counties in the highest tertile of percent minority population (27.9–99.3%), increases in objective social distancing were not associated with COVID-19 incidence (IRR = 0.89; 95%CI: 0.77–1.04) or mortality (IRR = 0.98; 95%CI: 0.76–1.27). In contrast, higher levels of objective distancing were protective for incidence (IRR = 0.67; 95%CI: 0.54–0.85) and mortality (IRR = 0.51; 95%CI: 0.36–0.71) in counties with the lowest percent of minority residents (0.0–9.8%). Similar patterns were observed with county median income, where objective social distancing was more protective in the wealthiest counties. Objective social distancing was protective in the highest and lowest tertiles of percent of residents aged 50 and older, but there was no association among the middle tertile. As expected, in counties with the highest levels of crowded households, objective social distancing measures were associated with smaller decreases in incidence or mortality, although this pattern was less clear when looking at the percentage of households with extreme crowding. Results were similar in models using objective social distancing measures for periods other than baseline and in Poisson models accounting for overdispersion (data not shown).

## 4. Discussion

In this analysis of the impacts of county-level objective social distancing and county-level COVID-19 incidence and mortality during the first wave of the pandemic in the United States, we observed that increases in objective social distancing were associated with decreased incidence and decreased mortality, even after adjusting for county-level sociodemographics, crowding, and obesity levels. Objective social distancing, however, was not equally protective in all counties. We observed that objective social distancing was less protective in counties with a larger minority population, lower median incomes, and more crowding. Social distancing was most beneficial in counties with the highest levels of median income.

Our results provide insights into the factors that may increase or decrease the efficacy of social distancing. The overall effectiveness of social distancing, however, is already clear. Our results for the entire United States are well in line with a recent modeling study of the correlations between daily cellphone-based mobility ratios and the rates of newly confirmed cases of COVID-19 in 25 United States counties with the highest case rates as of April 2020. Among these 25 counties, correlations between an 11-day lagged mobility ratio and the case–growth ratio ranged from 0.53 to 0.90, with an overall correlation of 0.71, indicating that increased distancing had a significant impact on case growth rates [5]. One analysis estimated, using national county-level data, that COVID-19 case counts would have been 35 times higher without any of the social distancing restrictions that were put in place in March and April of 2020 [20]. Social distancing, measured using Google’s community mobility reports, was estimated to have led to approximately 10,000 fewer deaths from COVID-19 in the Sao Paulo area of Brazil [21]. Social distancing policies were also shown to lead to 65% reductions in transmission in a study of 134 nations [4]. Restrictions on social distancing focus on public behavior in public spaces, but some of the most important microenvironments and activities that influence transmission likely occur outside of the public domain. We know that crowded homes, like crowded workplaces, provide multiple mechanisms for transmission through airborne and surface transfer. In Italy, one factor thought to contribute to COVID-19 cases was the spread driven by the high proportion of multigenerational households comprised of family structures such as a relatively higher mean age of leaving the parental household (30.1 years) compared to the European Union (26 years), frequent physical contact with extended families, and a relatively large proportion of the population (approximately 25%) being aged 65 years or older [22,23,24,25]. There is also a substantial body of literature demonstrating crowding and housing conditions as a key risk factor for the transmission of various infectious agents [26,27,28,29,30,31,32,33,34].

Social distancing is an effective tool because it addresses a mechanistic and modifiable pathway for viral transmission. However, the persistence and scale of health disparities are largely shaped by factors that are not easily modified for individuals, especially on a timescale that can contain a pandemic. Health disparities are often driven by mechanisms that are hidden from view. Housing, transportation modes, and occupational settings are critically important settings that are also known to have strong socioeconomic determinants. Our results illustrating the differences in social distancing efficacy across different types of counties are in line with a growing body of literature demonstrating vast disparities in COVID-19 infection and mortality rates. A recent analysis of death records during the first four months of 2020 in Massachusetts showed that, compared to previous years, mortality rates were significantly higher in communities with higher poverty, higher household crowding, higher percentage of populations of color, and higher racialized economic segregation [7]. These results have been confirmed in analyses including all U.S. counties [35].

Our study has several key limitations. First, our measures of outcome, exposures, and effect modifiers are all aggregated to the county level. This lack of granularity restricts our ability to observe associations and likely incorporates a substantial measurement error into our analyses. However, this would likely decrease our ability to detect patterns. Second, to draw straightforward comparisons, we chose not to incorporate data on social distancing after states began to relax social distancing rules. This limits the generalizability of our findings but allowed us to clearly examine the impacts of social distancing measures without accounting for time lags after reopening. It also allowed us to study the effects of social distancing before the wide onset of “pandemic fatigue.” Additional analyses using data spanning 2020–2021 and accounting for differences in the timing of and changes in stay-at-home orders and social distancing are warranted. Finally, we are unable to rule out the possibility that some other factor that varies at the county level may be driving the associations that we have observed. Other measures apart from social distancing have been implemented to mitigate the spread of infection including wearing masks [36], which also impacts the generalizability of our findings as different countries have utilized different public health control measures [37]. In addition, other variables that should be considered include testing coverage, contact tracing, quarantine of contacts, isolation of non-severe cases, social support, and environmental factors (e.g., humidity, temperature) that have been shown to be associated with the severity of illness and mortality due to COVID-19 [38]. Our models were generally robust to adjustment for many potential confounders, but given our ecological study design, this cannot be ruled out.

However, our study does have many strengths. We used multiple sources of publicly available data to assess the differential impact of social distancing on COVID-19 incidence and mortality for the entire U.S. Our results provide a framework for public health professionals to optimize social distancing guidance, and a way to identify communities that may require additional resources/strategies for transmission reduction. These may be more important as vaccination uptake varies across communities, to identify areas where social distancing is unlikely to be as efficient and vaccination uptake is low. Importantly, these results also demonstrate that known drivers of health disparities in the U.S. also lead to important gaps in prevention.

## 5. Conclusions

In conclusion, although we have demonstrated that in the first wave of the pandemic social distancing is an effective way to reduce COVID-19 infection and mortality, we have also shown that this strategy does not work evenly across populations. As local, state, and federal governments consider containment approaches and changes in approaches, it is important to determine if there are factors that may impact their efficacy across populations. A focus on developing effective strategies for populations experiencing crowding or with historical patterns of health disparities will be a key piece to reducing overall transmission and disease containment.

## Figures and Tables

**Table 1:** Association of each one-unit increase in county-level objective social distancing with the risk of county-level COVID-19 incidence or mortality in the United States through 29 April 2020.

COVID-19 Outcome	Model 1 ^a^ (IRR (95%CI))	Model 2 ^b^ (IRR (95%CI))	Model 3 ^c^ (IRR (95%CI))
Incidence	0.71 (0.63, 0.79) *p* ≤ 0.0001	0.75 (0.67, 0.84) *p* ≤ 0.0001	0.74 (0.66, 0.82) *p* ≤ 0.0001
Mortality	0.65 (0.55, 0.76) *p* ≤ 0.0001	0.70 (0.59, 0.83) *p* = 0.003	0.69 (0.58, 0.82) *p* = 0.001

^a^ Model 1 adjusted for county-level Hispanic ethnicity, minority race, percent aged 50 years and older, percent males, median household income, population density, obesity prevalence, and state-level cumulative COVID-19 testing rate. ^b^ Model 2 contains all variables in Model 1 and is additionally adjusted for county-level percent household crowding (>1 individual per room). ^c^ Model 3 contains all variables in Model 1 and is additionally adjusted for county-level percent extreme household crowding (>1.5 individuals per room).

**Table 2:** Effect modification of the association of each one-unit increase in county-level objective social distancing on the risk of county-level COVID-19 incidence or mortality in the United States through 29 April 2020.

Effect Modifier	Incidence ^a^ (IRR (95%CI))	Mortality ^a^ (IRR (95%CI))
Percent crowding		
Tertile 1: 0–1.45%	0.67 (0.54, 0.83)	0.64 (0.49, 0.83)
Tertile 2: 1.46–2.46%	0.73 (0.60, 0.90)	0.61 (0.47, 0.78)
Tertile 3: 2.47–49.35%	0.73 (0.59, 0.89)	0.76 (0.58, 1.01)
*p*-for-interaction	0.0003	0.002
Percent extreme crowding		
Tertile 1: 0–0.31%	0.73 (0.62, 0.86)	0.70 (0.52, 0.95)
Tertile 2: 0.32–0.66%	0.66 (0.51, 0.85)	0.70 (0.55, 0.89)
Tertile 3: 0.67–29.14%	0.70 (0.58, 0.85)	0.61 (0.45, 0.86)
*p*-for-interaction	0.03	0.54
Percent Hispanic		
Tertile 1: 0–2.66%	0.75 (0.59, 0.95)	0.73 (0.54, 1.01)
Tertile 2: 2.67–6.76%	0.69 (0.60, 0.80)	0.66 (0.50, 0.86)
Tertile 3: 6.77–99.07%	0.79 (0.68, 0.92)	0.62 (0.48, 0.79)
*p*-for-interaction	0.14	0.80
Percent minority		
Tertile 1: 0.31–9.75%	0.67 (0.54, 0.85)	0.51 (0.36, 0.71)
Tertile 2: 9.76–27.89%	0.71 (0.56, 0.91)	0.63 (0.49, 0.81)
Tertile 3: 27.90–99.27%	0.89 (0.77, 1.04)	0.98 (0.76, 1.27)
*p*-for-interaction	<0.0001	0.0004
Median household income		
Tertile 1: $20,188–$45,177	0.85 (0.73, 1.00)	0.80 (0.60, 1.06)
Tertile 2: $45,121–$54,661	0.78 (0.63, 0.95)	0.63 (0.49, 0.81)
Tertile 3: $54,691–$136,268	0.54 (0.44, 0.67)	0.46 (0.34, 0.63)
*p*-for-interaction	0.047	0.007
Percent aged 50 and older		
Tertile 1: 10.39–36.86%	0.76 (0.64, 0.91)	0.73 (0.57, 0.93)
Tertile 2: 36.87–41.35%	1.00 (0.79, 1.26)	1.09 (0.80, 1.46)
Tertile 3: 41.46–74.40%	0.72 (0.63, 0.83)	0.58 (0.44, 0.77)
*p*-for-interaction	0.002	0.002

^a^ Models adjusted for county-level Hispanic ethnicity, minority race, percent aged 50 years and older, percent males, median household income, population density, percent household crowding, obesity prevalence, and state-level cumulative COVID-19 testing rate as appropriate.

## Data Availability

All data are publicly available from the referenced sources (the COVID Tracking Project at: https://covidtracking.com/data (accessed on 25 April 2021). and the Unacast Social Distancing Scoreboard at: https://www.unacast.com/covid19/social-distancing-scoreboard (accessed on 25 April 2021). Statistical code is available upon request from the authors.
